# TzanckNet: a convolutional neural network to identify cells in the cytology of erosive-vesiculobullous diseases

**DOI:** 10.1038/s41598-020-75546-z

**Published:** 2020-10-27

**Authors:** Mehmet Alican Noyan, Murat Durdu, Ali Haydar Eskiocak

**Affiliations:** 1 Ipsumio B.V., High Tech Campus, 5656 AE Eindhoven, The Netherlands; 2 Department of Dermatology, Başkent University Medical School, Adana Dr. Turgut Noyan Application and Research Center, Adana, Turkey

**Keywords:** Medical imaging, Diseases, Health care, Medical research

## Abstract

Tzanck smear test is a low-cost, rapid and reliable tool which can be used for the diagnosis of many erosive-vesiculobullous, tumoral and granulomatous diseases. Currently its use is limited mainly due to lack of experience in interpretation of the smears. We developed a deep learning model, TzanckNet, that can identify cells in Tzanck smear test findings. TzanckNet was trained on a retrospective development dataset of 2260 Tzanck smear images collected between December 2006 and December 2019. The finalized model was evaluated using a prospective validation dataset of 359 Tzanck smear images collected from 15 patients during January 2020. It is designed to recognize six cell types (acantholytic cells, eosinophils, hypha, multinucleated giant cells, normal keratinocytes and tadpole cells). For 359 images and 6 cell types, TzanckNet made 2154 predictions. The accuracy was 94.3% (95% CI 93.4–95.3), the sensitivity was 83.7% (95% CI 80.3–87.0) and the specificity was 97.3% (95% CI 96.5–98.1). The area under the receiver operating characteristic curve was 0.974. Our results show that TzanckNet has the potential to lower the experience barrier needed to use this test, broadening its user base, and hence improving patient well-being.

## Introduction

A physician is expected to provide an accurate diagnosis. Accuracy, however, is hardly the only criterion for choosing the right diagnostic test. The cost has always been an important concern and is even more so today with rising healthcare costs in certain countries. Regulations add another dimension to consider. Many tests that are taken for granted in some clinics, might not be available in resource-constrained settings. Patient discomfort is yet another consideration. Therefore, it is hard to find a diagnostic test that checks all the boxes.

Tzanck smear test, a dermatopathological diagnostic tool first described in 1947^1^, costs less than a dollar, gives results in less than an hour, causes minimal patient discomfort, does not require anesthesia and is accurate^2^. It requires simple tools that almost all the clinics in the world can access—a microscope slide, an immersion oil, a scalpel, cytological stains and a microscope. With these advantages, one would expect it to be indispensable for dermatology and pathology.

Surprisingly, the reality is quite the opposite. Although this method can be used for the diagnosis of many erosive-vesiculobullous, tumoral and granulomatous diseases, its use is usually limited to herpes virus infections in daily dermatology practice^2^. In some clinics, this test is not used even for the diagnosis of herpes virus infections; instead patients receive local anesthesia followed by a skin biopsy^3^. Furthermore, when folliculitis patients are treated without cytological examination, patients with viral, parasitic and fungal folliculitis may receive unnecessary antibiotic treatments for years^4^. One of the reasons for this is the lack of experience in using this test. Efforts have been made in the past decade to reintroduce Tzanck smear test^5–10^ which has resulted in revived interest^11–13^. Still, this diagnostic tool is nowhere near its full potential. The main textbooks of dermatopathology do not include dedicated sections for cytology or Tzanck smear^14,15^.

Artificial intelligence has long been used to tackle challenges in healthcare^16–19^, with growing attention in the last years. It has found wide ranging roles from automating menial tasks^20^ to optimizing medical resources^21^. The spotlight, however, is on deep learning models showing expert level performance on certain medical skills—even surpassing humans in some instances^22–25^. Analyzing medical images constitute an important part of these skills^26^ because the development of deep learning has been strongly tied to image processing^27^. Artificial intelligence models have been used in dermatology^19,23,28–31^, but, to the best of our knowledge, they have never been applied to cutaneous cytology or evaluating Tzanck smear findings.

**Figure 1:**
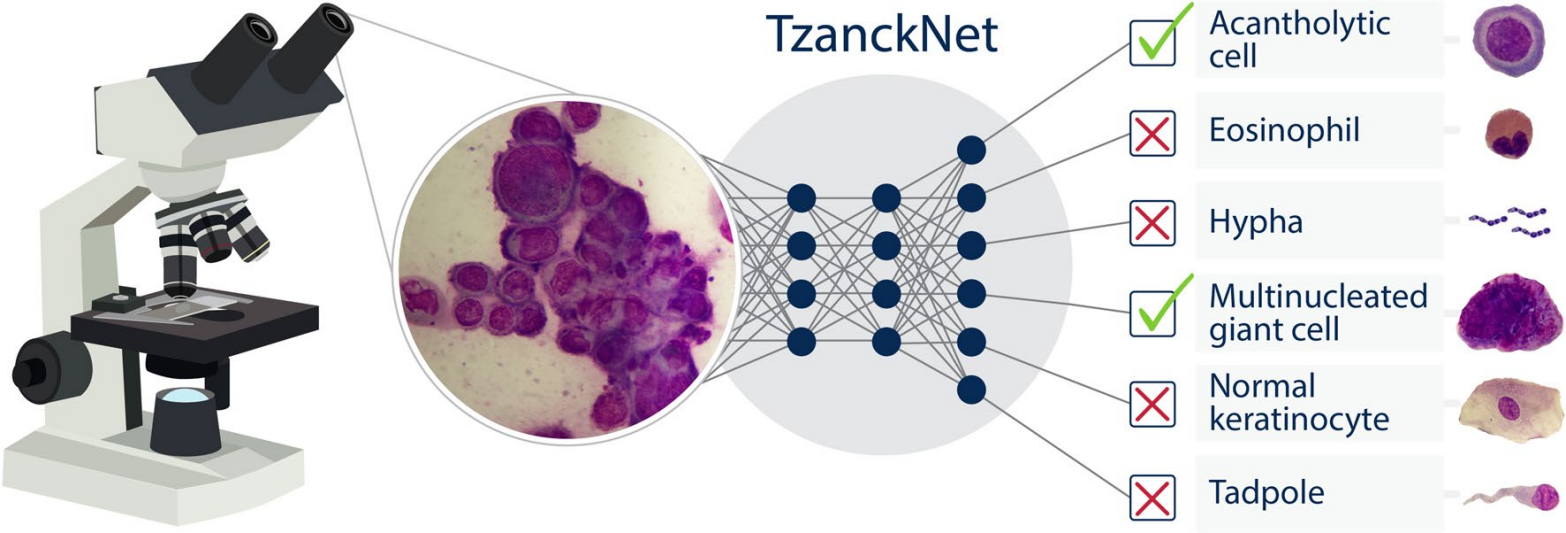
A schematic showing how the proposed TzanckNet works. TzanckNet accepts a Tzanck smear image as an input and outputs which cell types among six cell types are present and absent in the image. As an example, a Tzanck smear image from a patient with herpetic infection goes into the TzanckNet and the network predicts that there are acantholytic and multinucleated giant cells in the image, the remaining four cell types does not exist in the image. On the right-hand side of the figure, an example image from each cell type is presented.

The aim of this study was to develop a deep neural network, called TzanckNet, for recognizing cells in Tzanck smear test findings of erosive-vesiculobullous diseases. It was designed to recognize six cell types related to diseases such as herpetic infections, pemphigus, and spongiotic dermatitis (Fig. 1). The model was developed using a retrospective dataset and validated clinically using a prospective dataset.

## Methods

### Datasets

For developing the TzanckNet, we used the cytology archive of Department of Dermatology, Başkent University Medical School, Adana Dr. Turgut Noyan Application and Research Center. This study was approved by Başkent University Institutional Review Board (Project no: KA19/401). We confirm that this work was performed in accordance with relevant guidelines/regulations, and informed consent was obtained from all participants or their legal guardians. There were 2260 Tzanck smear images from erosive-vesiculobullous diseases (pemphigus, herpetic infection, impetigo, Hailey-Hailey disease, contact dermatitis, vesiculobullous dermatophytic infection) collected between December 2006 and December 2019. This dataset is called the *development dataset*. It was used to train and fine tune the model. Afterwards, the finalized model was evaluated using a separate dataset that was collected during January 2020 in the same clinic. It contained 359 images from patients that were not in the development dataset. This is called the *validation dataset*. The images in this dataset were obtained from 15 patients (9 females, 6 males) with ages ranging from 2 to 68 years (34 years on average).

We would like to note that most studies use retrospective datasets and split these into development and validation datasets. However, such approaches can result in information leakage between the two sets. To avoid such issues, we collected our validation set after the TzanckNet was trained and finalized. This limited the number of available patients. We considered 15 patients as adequate for testing since the number of images (359) is more important than the number of patients (15) for this study as cytological properties of cells are not affected by the patient’s demographic properties and two images from the same patient might look very different.

### Dataset preparation

Cytological specimens were stained with May-Grünwald-Giemsa (Bio-Optica), examined microscopically and photographed with a digital camera. Cytological photos taken at × 1000 magnification. Two dermatologists (M.D. and A.H.E.) prepared the dataset with two different microscopes and with two different digital cameras. There were no missing data in the datasets. Images with artifacts (overlapping cells, scratches etc.) were not removed from the dataset. Only images that were blurry at a level that would make them useless in a clinical setting were discarded and the samples were imaged again. To avoid selection bias, both positive and negative regions of the smears were imaged. Two types of regions were considered as negative; regions with normal keratinocytes and regions that are positive for certain cells are negative for the remaining cell types. Images were labeled, de-identified and given to the data scientist (M.A.N.).

### Reference standard

Images were labeled by two dermatologists (M.D. and A.H.E.). M.D. has 13 years and A.H.E. has 2 years of previous cutaneous cytology experience. Labels were decided by adjudication^32^ i.e. disagreements were resolved through discussion. The image was labeled positively for a cell only if that cell was entirely present in the image.

### Machine learning model development

A deep learning library, fastai^33^, was used to develop the TzanckNet. It is a convolutional neural network (ResNet-50), pretrained on ImageNet. Then, the final classification layer was replaced with six output nodes and retrained. Six output nodes in the final layer correspond to the six cell types we chose our model to recognize: acantholytic cells, eosinophils, hypha, multinucleated giant cells, normal keratinocytes and tadpole cells (Fig. 1). An image can contain any combination of these cells; therefore, TzanckNet was designed such that it can output any number/combination of cells for one input image. Each output node, outputs a value between 0 and 1, corresponding to the probability of that cell existing in the input image. Depending on the discrimination threshold this probability is converted to 1 or 0 indicating whether the cell type exists in the image or not. For this study, the discrimination threshold was kept constant as 0.5, except for plotting the receiver operating characteristic curve. The development dataset was used at this stage, for training and tuning the model. TzanckNet can process any number of images at once. Therefore, it is suitable for a single microscopy image taken from a region of interest as well as a whole-slide image split into tiles.

### Statistical analysis

Discrimination and calibration performance were used to assess the TzanckNet. The following metrics were used for discrimination: accuracy, sensitivity, specificity, positive predictive value, negative predictive value and F_1_ score with a discrimination threshold of 0.5. Additionally, receiver operating characteristic curve (ROC curve) was plotted to evaluate the model at different discrimination threshold values. Area under this curve (AUC) was calculated and reported as well. Calibration curve was used to evaluate how well predicted probabilities approximated the actual event probabilities. The validation dataset was used at this stage for evaluating the model. Finally, TzanckNet predictions on selected images were demonstrated.

## Results

Using a convolution neural network, we created a machine learning model called TzanckNet for recognizing six cell types in a Tzanck Smear image. An image can contain multiple cell types, for this reason we designed the model such that it can output more than one cell type. Given one input image, the model outputs six probabilities, corresponding to the predicted probabilities of the existence of six cell types. We fixed a discrimination threshold of 0.5 such that if the predicted probability for any cell type was above this threshold, the model predicted that this cell type existed in the image. These predictions were compared to the reference standard for evaluating the model performance.

The TzanckNet, trained and tuned on the development set, was prospectively validated using 359 images collected in a real-world setting. For each image it made six predictions hence it made 2154 predictions in total. The overall accuracy of the TzanckNet on this dataset was 94.3% (95% CI 93.4–95.3), the sensitivity was 83.7% (95% CI 80.3–87.0) and the specificity was 97.3% (95% CI 96.5–98.1). Results of the other discrimination metrics and performance for each cell are given in Table 1.

**Table 1:** Discrimination metrics of the TzanckNet on the validation dataset that contains 359 Tzanck smear findings.

	Accuracy	Sensitivity	Specificity	PPV	NPV	AUC	F_1_ score (%)
Acantholytic cell	315/359 (87.7%)	118/139 (84.9%)	197/220 (89.5%)	118/141 (83.7%)	197/218 (90.4%)	0.954	84.3
Eosinophil	324/359 (90.3%)	24/59 (40.7%)	300/300 (100%)	24/24 (100%)	300/335 (89.6%)	0.918	57.8
Hypha	352/359 (98.1%)	39/39 (100%)	313/320 (97.8%)	39/46 (84.8%)	313/313 (100.0%)	0.999	91.8
Multinucleated giant cell	353/359 (98.3%)	109/114 (95.6%)	244/245 (99.6%)	109/110 (99.1%)	244/249 (98.0%)	0.998	97.3
Normal keratinocyte	359/359 (100%)	47/47 (100%)	312/312 (100%)	47/47 (100%)	312/312 (100%)	1	100%
Tadpole cell	329/359 (91.6%)	52/67 (77.6%)	277/292 (94.9%)	52/67 (77.6%)	277/292 (94.9%)	0.959	77.6%
Overall	2032/2154 (94.3%)	389/465 (83.7%)	1643/1689 (97.3%)	389/435 (89.4%)	1643/1719 (95.6%)	0.974	86.4%

*PPV* positive predictive value, *NPV* negative predictive value, *AUC* area under the receiver operating characteristic curve.

**Figure 2:**
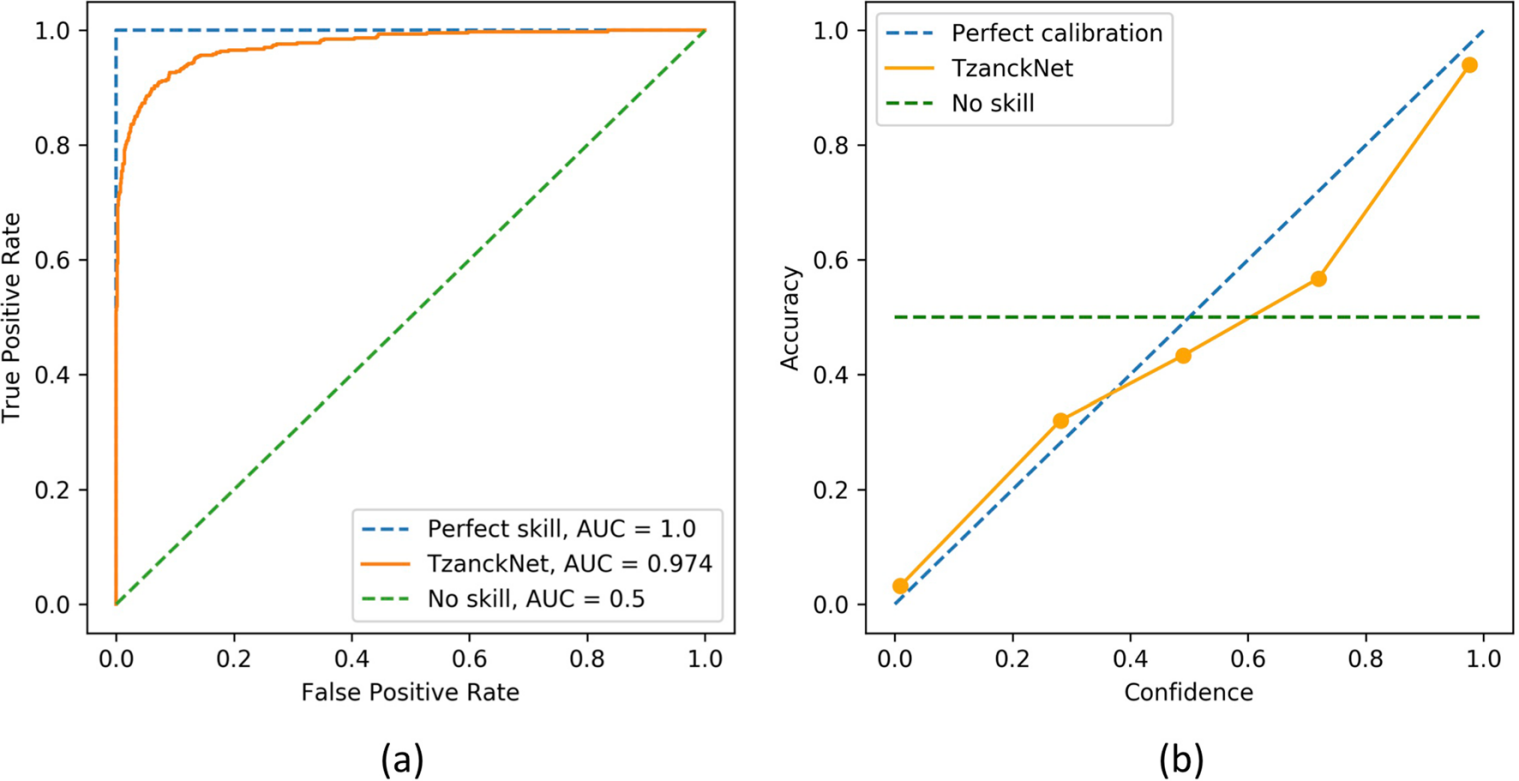
(**a**) Receiver operating characteristic (ROC) and (**b**) calibration curves of the TzanckNet on the validation set.

In order to visualize model performance across different discrimination thresholds, overall receiver operating characteristic curve of the network is given in Fig. 2a. The area under this curve (AUC) was 0.974. For assessing the performance of the predicted probabilities, calibration curve is given in Fig. 2b.

**Figure 3:**
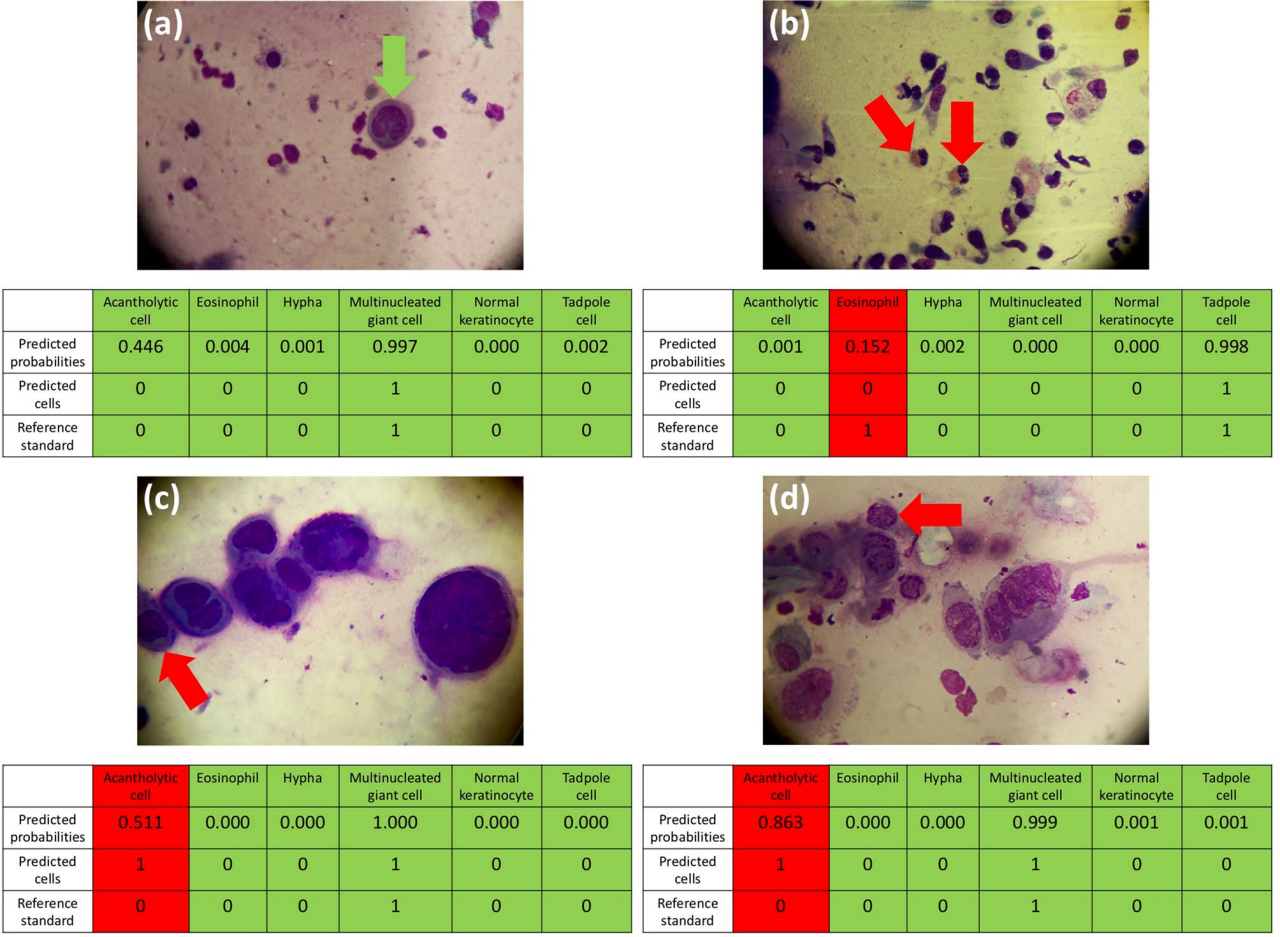
TzanckNet predictions and the corresponding reference standards for four selected images. For each cell type and image, TzanckNet predicts the probability of that cell type being present in the image. Probabilities are then converted to decisions of absence (0) or presence (1) of that cell, using a discrimination threshold of 0.5. Decisions matching with the reference standards are marked with green, and with red otherwise. The red arrows indicate the cells that are related to the false predictions. The green arrow indicates a multinucleated giant cell.

TzanckNet performance on selected Tzanck smear findings are given in Fig. 3. Figure 3a contains a multinucleated giant cell (arrow). At first sight during labeling, it seemed like the cell had one circular nucleus and was hence labeled as an acantholytic cell. Upon further examination, it was realized that the cell actually had three nuclei instead of one and the label was changed to a multinucleated giant cell. The TzanckNet recognized that there was as a multinucleated giant cell, but it also gave a 44.6% probability for the existence of an acantholytic cell. Two eosinophils can be seen in Fig. 3b (arrows), however, TzanckNet didn’t recognize them. In Fig. 3c, only some part of an acantholytic cell (arrow) is visible inside the image therefore the image was labelled as having no acantholytic cell in the image. TzanckNet predicted that there was an acantholytic cell, but it was not confident (51.1% predicted probability). Figure 3d contains an atypical acantholytic cell (arrow). It is atypical because the perinuclear halo is not evident and in some parts cytoplasm is not discernible. Therefore, the image was labeled as not having an acantholytic cell. The TzanckNet, however, detected that there is an acantholytic cell in this image, and it was 86.3% confident.

## Discussion

Tzanck smear test is an inexpensive test that can provide rapid diagnosis of many dermatological diseases. Interest in Tzanck smear test has increased in recent years. Nevertheless, in most clinics, this simple test is either not performed or is only used to diagnose a few diseases, mainly due to lack of experience. In a dermatology clinic where 75 patients for whom no diagnosis could be established via face-to-face clinical examinations and cytological evaluations, telemedicine was used to overcome this issue^34^. Although this is a reasonable solution, the necessary expertise is still scarce, scheduling is necessary, and one can only examine a limited number of images during a session. In this work we demonstrated that TzanckNet can identify six cell types in Tzanck smear findings. It can analyze hundreds of images in a minute with high accuracy.

The overall performance of the TzanckNet shows that deep learning has the potential to aid dermatologists for analyzing Tzanck smear findings. It was able to recognize cells with high accuracy. Moreover, the calibration performance shows that the predicted probabilities can also help with the interpretation of images. For example, if a cell type exists with 99% estimated probability the network is almost certain that this cell is present in the image whereas if the estimated probability is 70% the network tends to predict that this cell is present but not confidently. If the network is not confident about its predictions, additional images can be taken.

Examining individual predictions can provide a better understanding of the model’s strengths and limitations hence indicating ways to improve it furthermore. For example, TzanckNet was able to recognize correctly a multinucleated cell that looks similar to an acantholytic cell (Fig. 3a). Looking at Table 1, low sensitivity on eosinophils draws attention. Specificity being 100% indicates that all of the errors for eosinophils were false negatives, there were no false positives. Individual examination of the images that resulted in false negatives revealed that they had less than five eosinophils on average whereas the examination of true positives revealed that they had more than ten eosinophils on average. One of the images that resulted in a false negative eosinophil can be seen in Fig. 3b. Indeed, it contains only two eosinophils (arrows). This suggests that for TzanckNet to recognize eosinophils, they should be abundant in the image. Figure 3c,d indicates model performance may not be ideal for cells that are partially in the image or atypical cells.

The most common use of Tzanck smear test is the diagnosis of herpetic infection^7,35^. Herpetic infections may be primary or develop on other dermatoses. Early diagnosis of herpetic infections is very important in terms of early treatment and prevention of the spread of the disease. When Tzanck smear is not performed, patients receive unnecessary treatment or more complex diagnostic methods such as polymerase chain reaction or histopathological examinations are requested^2^. Tzanck smear test is cheaper and faster than both of these methods^4^. The characteristic finding of herpetic infection in the Tzanck smear test is acantholytic cells with multinuclear giant cells^2^. Another disease that is related to these cells is pemphigus. It is a rare and severe autoimmune disease that can cause mortality. Tzanck smear test is not only important in early diagnosis of the disease but also in the diagnosis of recurrent lesions. A definitive diagnosis of pemphigus requires histopathological examination and immunofluorescence test. However, it is not possible to take a biopsy each time for recurrent lesions. Herpetic infection and candidiasis should be excluded ruled out especially in newly developed lesions resistant to corticosteroid treatment in oral mucosa. If this test is not performed, patients will receive unnecessary steroid treatment, which may lead to sepsis^36^. In pemphigus patients, unlike herpetic infections, acantholytic cells without multinuclear giant cells are observed in cytological examination^2,9^. In this study, TzanckNet showed that it was able to recognize acantholytic and multinuclear giant cells demonstrating its diagnostic potential for suspected herpetic infection and pemphigus.

A few tadpole cells are observed in all vesiculobullous diseases. However, numerous tadpole cells are characteristic cytologic findings of spongiotic dermatitis^37^ and eczema. Tzanck smear is used for two purposes in eczema patients. The first is to distinguish eczema from other acantholytic diseases and the second is to detect bacterial, fungal and viral infections. Tzanck smear test performed for both purposes prevent unnecessary treatment. If the test is not done, infections can spread easily with steroid treatments used for eczema. Accuracy of the model on tadpole cells was 91.6%, however it should be noted that the specificity was high, but sensitivity was low suggesting that the model tends to give false negatives.

Cutaneous eosinophilic infiltration may develop due to various causes such as infectious, inflammatory and neoplastic diseases. Histopathology is often used in the differentiation of these diseases. However, cytological examination has been used in only a few studies focused on the utility of cytology in some eosinophilic diseases. Besides detecting eosinophils, cytology can also show the various infectious etiologic agents and distinguish some inflammatory diseases. If eosinophils are present, bacterial and fungal structures should be investigated. For example, *Pseudomonas aeruginosa* and *Staphylococcus aureus* can cause eosinophilic folliculitis^38^. Additionally, for the cytological examination of erosive-vesiculobullous lesions, neutrophils alone do not provide information, but eosinophils can. For example, in erosive lesions of pemphigus patients, neutrophils and cocci are observed, mostly due to secondary infections. For pustular lesions, eosinophils alone are the sign of eosinophilic pustular folliculitis. In a cytologic examination performed from an ulcerated lesion, the presence of abundant eosinophils without other cytologic findings is a sign of eosinophilic ulceration^2^. The performance of TzanckNet on eosinophils and hypha suggests that the network can also be utilized as a decision support tool for diagnosing aforementioned diseases.

The overall performance of the TzanckNet demonstrates its strong diagnostic potential. We would like to emphasize that we propose this model as a clinical decision support system to improve physician accuracy and efficiency in the clinical workflow, not as a replacement or a tool that can directly output a diagnosis. It has been shown that human–machine combination works better than either alone^39–41^. While it is possible to make a machine learning model that can directly output a diagnosis, we consider such black box approaches to be unsuitable for high stakes decisions like medical diagnosis. Using the output of the TzanckNet combined with other possible findings, the physicians in charge can reach a validated and verified diagnosis. For example, eosinophils are observed in many diseases but if observed in a pustular lesion without infectious agents indicates eosinophilic pustular folliculitis. Additionally, TzanckNet can be used to train dermatologists on Tzanck smear test.

### Limitations

Like all methods/models, explicitly reporting known limitations of TzanckNet is crucial for clinicians and researchers who want to use and improve it. The model was trained using Tzanck smear findings of patients from Turkey, stained with May-Grünwald-Giemsa and taken with × 1000 magnification. The performance of the model on other populations, sample preparation procedures and imaging systems remains unknown at this point. Model performance on cells that are atypical or that overlap or appear partially in the image may not be ideal. Finally, yet importantly, the model can recognize which cell types exist in an image, but it cannot localize the cells. Localizing the cells has many advantages such as being able to count the cells and being able to say precisely which cells in the image are recognized and what their types are. Two major improvements are needed to realize the full potential of the TzanckNet: the first is to retrain and validate the model with other populations, stains, and magnifications for better generalization, and the second is to add object detection to the model for localizing the cells.

Nevertheless, this study showed that TzanckNet can analyze the cytological findings of erosive-vesiculobullous diseases accurately. The model can be extended for analyzing granulomatous and tumoral diseases.

## Conclusions

Tzanck smear test is a valuable but underappreciated diagnostic tool. This work introduced TzanckNet, a machine learning model that can analyze Tzanck smear findings with high accuracy. It can be used as a clinical decision support system as well as a training tool for new physicians. It has the potential to spread the use of Tzanck smear test, decrease the number of biopsies, prevent unnecessarily long antibiotic treatments, help early diagnoses for fatal diseases, decrease costs, and thus improve patient well-being.

## References

[1] Tzanck AL (1947). cyto-diagnostic immédiat en dermatologie. Press Med..

[2] Durdu M (2019). Cutaneous Cytology and Tzanck Smear Test.

[3] Horn TD (2008). Commentary: heading the wrong way. The disappearing Tzanck smear. J. Am. Acad. Dermatol..

[4] Durdu M, Ilkit M (2013). First step in the differential diagnosis of folliculitis: cytology. Crit. Rev. Microbiol..

[5] Ruocco V, Ruocco E (1999). Tzanck smear, an old test for the new millennium: when and how. Int. J. Dermatol..

[6] Gupta LK, Singhi MK (2005). Tzanck smear: a useful diagnostic tool. Indian J. Dermatol. Venereol. Leprol..

[7] Durdu M, Baba M, Seçkin D (2008). The value of Tzanck smear test in diagnosis of erosive, vesicular, bullous, and pustular skin lesions. J. Am. Acad. Dermatol..

[8] Kelly B, Shimoni T (2009). Reintroducing the Tzanck smear. Am. J. Clin. Dermatol..

[9] Durdu M, Baba M, Seçkin D (2009). More experiences with the Tzanck smear test: cytologic findings in cutaneous granulomatous disorders. J. Am. Acad. Dermatol..

[10] Ruocco E, Brunetti G, Del Vecchio M, Ruocco V (2011). The practical use of cytology for diagnosis in dermatology. J. Eur. Acad. Dermatol. Venereol..

[11] Yaeen A, Ahmad Q, Farhana A, Shah P, Hassan I (2015). Diagnostic value of Tzanck smear in various erosive, vesicular, and bullous skin lesions. Indian Dermatol. Online J..

[12] Zhou T, Fang S, Li C, Hua H (2016). Comparative study of indirect immunofluorescence, enzyme-linked immunosorbent assay, and the Tzanck smear test for the diagnosis of pemphigus. J. Oral Pathol. Med..

[13] Panwar H (2017). Diagnostic utility and pitfalls of Tzanck smear cytology in diagnosis of various cutaneous lesions. J. Cytol..

[14] Elder D (2014). Lever’s Histopathology of the Skin.

[15] Barnhill R (2019). Dermatopathology.

[16] Bartels PH, Weber JE, Duckstein L (1988). Machine learning in quantitative histopathology. Anal. Quant. Cytol. Histol..

[17] Stiell IG (1992). A study to develop clinical decision rules for the use of radiography in acute ankle injuries. Ann. Emerg. Med..

[18] Wolberg WH, Street WN, Heisey DM, Mangasarian OL (1995). Computer-derived nuclear features distinguish malignant from benign breast cytology. Hum. Pathol..

[19] Dreiseitl S (2001). A comparison of machine learning methods for the diagnosis of pigmented skin lesions. J. Biomed. Inform..

[20] Lin SY, Shanafelt TD, Asch SM (2018). Reimagining clinical documentation with artificial intelligence. Mayo Clin. Proc..

[21] Horng S (2017). Creating an automated trigger for sepsis clinical decision support at emergency department triage using machine learning. PLoS ONE.

[22] Gulshan V (2016). Development and validation of a deep learning algorithm for detection of diabetic retinopathy in retinal fundus photographs. JAMA.

[23] Esteva A (2017). Dermatologist-level classification of skin cancer with deep neural networks. Nature.

[24] Chilamkurthy S (2018). Deep learning algorithms for detection of critical findings in head CT scans: a retrospective study. Lancet.

[25] Tomašev N (2019). A clinically applicable approach to continuous prediction of future acute kidney injury. Nature.

[26] Litjens G (2017). A survey on deep learning in medical image analysis. Med. Image Anal..

[27] Lecun Y, Bengio Y, Hinton G (2015). Deep learning. Nature.

[28] Fujisawa Y (2019). Deep-learning-based, computer-aided classifier developed with a small dataset of clinical images surpasses board-certified dermatologists in skin tumour diagnosis. Br. J. Dermatol..

[29] Tschandl P (2019). Expert-Level diagnosis of nonpigmented skin cancer by combined convolutional neural networks. JAMA Dermatol..

[30] Brinker TJ (2019). A convolutional neural network trained with dermoscopic images performed on par with 145 dermatologists in a clinical melanoma image classification task. Eur. J. Cancer.

[31] Han SS (2020). Keratinocytic skin cancer detection on the face using region-based convolutional neural network. JAMA Dermatol..

[32] Krause J (2018). Grader variability and the importance of reference standards for evaluating machine learning models for diabetic retinopathy. Ophthalmology.

[33] Howard J, Gugger S (2020). Fastai: a layered API for deep learning. Information.

[34] Durdu M, Harman M (2016). Diagnostic value of telecytology in tertiary teledermatological consultation: a retrospective analysis of 75 cases. Int. J. Dermatol..

[35] Solomon AR, Rasmussen JE, Varani J, Pierson CL (1984). The Tzanck smear in the diagnosis of cutaneous herpes simplex. JAMA.

[36] Kalajian AH, Callen JP (2007). Atypical herpes simplex infection masquerading as recalcitrant pemphigus vulgaris. Australas. J. Dermatol..

[37] Pariser RJ (1983). Diagnosis of spongiotic vesicular dermatitis by Tzanck smear: the “tadpole cell”. J. Am. Acad. Dermatol..

[38] Nomura T, Katoh M, Yamamoto Y, Miyachi Y, Kabashima K (2016). Eosinophilic pustular folliculitis: a proposal of diagnostic and therapeutic algorithms. J. Dermatol..

[39] Steiner DF (2018). Impact of deep learning assistance on the histopathologic review of lymph nodes for metastatic breast cancer. Am. J. Surg. Pathol..

[40] Sayres R (2019). Using a deep learning algorithm and integrated gradients explanation to assist grading for diabetic retinopathy. Ophthalmology.

[41] Patel BN (2019). Human–machine partnership with artificial intelligence for chest radiograph diagnosis. NPJ Digit. Med..

